# Analysis of gastrin‐17 and its related influencing factors in physical examination results

**DOI:** 10.1002/iid3.993

**Published:** 2023-10-27

**Authors:** Junchao Zeng, Yan Shen, Sanping Xu, Rui Yang

**Affiliations:** ^1^ Health Management Center, Union Hospital, Tongji Medical College Huazhong University of Science and Technology Wuhan Hubei China

**Keywords:** gastrin‐17, *Helicobacter pylori*, inflammatory factors, pepsinogen

## Abstract

**Background:**

To analyze the difference of serum gastrin‐17 (G17) level in healthy people with different sex, age, and body mass index (BMI), to explore the correlation between G17 and pepsinogen, and to study the influences of *Helicobacter pylori* (*H. pylori*) infection and various inflammatory factors on G17 secretion level.

**Methods:**

A total of 531 subjects who received physical examination in our center from April 2019 to December 2019 were enrolled in the study. All subjects were tested for G17, pepsinogen I (PGI), pepsinogen II (PGII), PGI/PGII ratio (PGR), *H. pylori*, serum amyloid A (SAA), C‐reactive protein (CRP) and erythrocyte sedimentation rate (ESR). The difference of G17 secretion in different subjects and its correlation with PG were analyzed to investigate *H. pylori* infection and expound the effects of inflammatory indicators on G17.

**Results:**

There was no significant difference in G17 secretion level in people with different sex, age and BMI (*p* > .05). G17 positively correlated with PGI and PGII, but negatively correlated with PGR. The G17 level of *H. pylori*‐positive subjects was 10.16 ± 12.84, and prominently higher than that of *H. pylori*‐negative subjects (3.27 ± 6.65). SAA and *H. pylori* infection were the greater risk factors for G17 abnormality among various indicators. CRP and ESR had no effect on G17 abnormality.

**Conclusions:**

G17 secretion is closely related to PG and *H. pylori*. Combined screening contributes to early screening of gastrointestinal diseases in normal people or groups at high risk for gastric cancer, but the influence of inflammatory indicators on G17 should be excluded to improve the reliability of the results.

## 1 INTRODUCTION

Gastric cancer is a common digestive tract malignant tumor, ranking top in terms of incidence and mortality rates among global malignant tumors.^1^ It is the final consequence of the progression of precancerous lesions, including non‐atrophic gastritis (AG), AG, intestinal metaplasia (IM) and dysplasia. Epidemiological studies showed that the 5‐year survival rate of early gastric cancer is as high as 90%, while that of advanced gastric cancer is only 25%.^2^ Therefore, early screening, diagnosis and treatment are vital avenues to reduce the mortality of gastric cancer.

Compared with the markers in tissues, serological markers are superior due to their less invasiveness, easy availability, lower cost and shorter time consumption. Currently, available serological markers associated with gastric diseases include Pepsinogen I (PGI), Pepsinogen II (PGII), PG I/PG II (PGR), gastrin‐17 (G17) and anti‐*Helicobacter pylori* (*H. pylori* IgG). These serological markers may be conducive to identifying people with precancerous lesions who should undergo gastroscopy.^3^ Notably, these gastric biomarkers have been available to perform the serological biopsy of gastric mucosa to detect gastric cancer and precancerous lesions for more than 20 years. However, current studies have focused not only on PGI, PGI/II ratio and *H. pylori* IgG, but also other biomarkers such as G17 and PGII.

G17 is closely related to inflammation, immunity, invasion and metastasis of gastric cancer cells, and is lowly expressed in healthy people. When the pathological changes of gastritis, gastric cancer, etc. occur, G17 expression level will increase significantly.^4^ However, throughout the progression of gastric disease, the diagnostic intensity of serum G17 for AG showed a decreasing trend in the later stage.^5^ Intriguingly, the increase or decrease of G17 expression can indicate the occurrence risk of gastric cancer.^6^ G17 itself is not a direct inducer of gastric cancer, but it can promote some carcinogenic factors, activate multiple signal transduction pathways, exert antiapoptotic and antiinflammatory effects, and induce gastric acid secretion, thus leading to tumorigenesis.^7^ This study aims to analyze G17 and its possible influencing factors in healthy people, so as to fathom out the secretory characteristics and application value of G17 in healthy people.

## 2 MATERIALS AND METHODS

### 2.1 Participants

A total of 531 healthy subjects (307 males and 224 females; aging 22–78 years) who received physical examination in the Healthcare Department Center of Wuhan Union Hospital from April 2019 to December 2019 were selected by continuous and effective sampling method, and approved by the Ethics Committee of the hospital (No. S426). In addition to routine physical examination, G17, PGI, PGII, PGR, *H. pylori* typing, C‐reactive protein (CRP), erythrocyte sedimentation rate (ESR) and serum amyloid A (SAA) were detected. None of these subjects was diagnosed with acute and chronic inflammatory disease, liver and gallbladder disease, diabetes, blood disease, kidney disease, rheumatic immune disease, tumor, etc. Groups were constructed according to age, body mass index (BMI), and *H. pylori* typing.

### 2.2 Serological test

About 3 mL fasting venous blood was collected and centrifuged to obtain serum. The serum PGI, PGII, PGR, and G17 were quantitatively detected by enzyme‐linked immunosorbent assay. The testing equipment was procured from BIOHIT Healthcare (Hefei) Co., Ltd and Wuhan Yolins Technology Trade Co., Ltd. As per the instructions of the kit, PGI <70 ng/mL concurrently with PGR <3 was defined as PG‐positive, and G17 <1 or >15 pmol/L was regarded as G17‐positive.

### 2.3 ^13^C breath test

Subjects underwent the ^13^C breath test early in the morning on an empty stomach. The test was implemented using the kit produced by Shenzhen Zhonghe Headway Bio‐Sci & Tech Co., Ltd., and the HCBT‐02 ^13^C breath test instrument. The positive value of *H. pylori* infection was 4.0 times higher than the reference value.

### 2.4 Inflammatory index detection

CRP was measured by immunofluorescence quantitative detection using an I‐CHROMA Rester immunofluorescence analyzer (Boditech Med Inc.). ESR was calculated according to the Westergren method. SAA was detected with a SIEMENS BN prospect specific protein analyzer as well as matching reagents in strict accordance with the operation manual.

### 2.5 Statistical method

SPSS19.0 statistical software was used for analysis. Comparison between two groups was completed by *t* test, while contrast among multiple groups was achieved through one‐way analysis of variance. The correlation analysis was performed by Spearman rank correlation test, with the calibration level of 0.01. The simple Logistic regression analysis was used to evaluate the influencing factors. A *p* < .05 was considered to be statistically significant.

## 3 RESULTS

### 3.1 G17 secretion in subjects with different sex, age, and BMI

Serum G17 level was slightly higher in females (6.38 ± 9.85) than in males (5.32 ± 10.11). The G17 secretion level in the different age groups (≤30, 31–40, 41–50, 51–60, 61–70, ≥70 years old) was 5.01 ± 10.68, 4.17 ± 8.13, 4.81 ± 7.40, 7.66 ± 12.73, 6.58 ± 11.11, and 8.65 ± 6.15, respectively, with no significant difference among them. Moreover, G17 secretion level did not differ in the three BMI groups (<24, 5.35 ± 8.74; 24–27.9, 6.60 ± 12.35; ≥28, 5.70 ± 7.84). Collectively, the G17 secretion level was not statistically different in sex, age, and BMI groups (*p* > .05) (Table 1).

**Table 1:** G17 secretion in subjects with different sex, age, and BMI.

Groups	*N*	_x ± s	*t*/*F*	*p*
Sex				
male	307	5.32 ± 10.11	1.204	.299
female	224	6.38 ± 9.85		
Age				
≤30	39	5.01 ± 10.68	2.178	.055
31–40	86	4.17 ± 8.13		
41–50	198	4.81 ± 7.40		
51–60	164	7.66 ± 12.73		
61–70	39	6.58 ± 11.11		
≥70	5	8.65 ± 6.15		
BMI				
<24	174	5.35 ± 8.74	0.606	.546
24–27.9	146	6.60 ± 12.35		
≥28	58	5.70 ± 7.84		

Abbreviation: BMI, body mass index.

### 3.2 Analysis of influencing factors of G17

The risk factors of G17 secretion abnormality were analyzed, including sex, age, *H. pylori* infection, CRP, SAA, and ESR. Based on the data, SAA ( *p* = .016) and *H. pylori* infection ( *p* = .017) were the major risk factors for G17 abnormality. Other factors had no effect on the secretion of G17 (Table 2, *p* > .05).

**Table 2:** Analysis of influencing factors of G17.

Factors	HR	95% CI	*p*
Sex	1.098	0.716, 1.685	.699
Ages	1.018	0.995, 1.040	.121
*H. pylori* positive	1.713	1.100, 2.688	.017
CRP	0.552	0.285, 1.071	.079
SAA	2.692	1.202, 6.028	.016
ESR	1.698	0.712, 4.051	.233

Abbreviations: CI, confidence interval; CRP, C‐reactive protein; ESR, erythrocyte sedimentation rate; HR, hazard ratio; SAA, serum amyloid A.

### 3.3 Secretion of G17 in *H. pylori*‐infected subjects

The secretion of G17 in *H. pylori*‐infected subjects (10.16 ± 12.84) was remarkably higher than that in uninfected subjects (3.27 ± 6.65) (Table 3, *p* < .05).

**Table 3:** Secretion of G17 in subjects with or without *H. pylori* infection.

*H. pylori*	*N*	_x ± s	*t*	*p*
Negative	318	3.27 ± 6.65	6.356	.000
Positive	159	10.16 ± 12.84		

### 3.4 Correlation analysis between G17 and PG

G17 and PG were markedly correlated (*p* = .000). G17 positively correlated with PGI and PGII (*r* = .322 and .452), but negatively correlated with PGR (*r* = −.367) (Table 4).

**Table 4:** Correlation analysis between G17 and PG.

Serum markers	Median	*r*	*p*
PGI	57.49 (3.3–17.65)	.322	.000
PGII	9.80 (2.5–41.3)	.452	.000
PGR	6.63 (0.37–16.83)	−.367	.000

## 4 DISCUSSION

As a gastrointestinal hormone, G17 can not only maintain the integrity of digestive tract structure, but also regulate the function of digestive tract.^6^ Besides, G17 also has biological functions of promoting proliferation and inhibiting apoptosis.^8^ The changes of serum G17 level can reflect the state of gastric mucosal lesions, so it can serve as a screening index for gastric cancer and precancerous diseases.^9^ In our study, as with other researches, G17 level was not different in different sex, age and BMI.^10^ However, several previous studies have reported that G17 level is remarkably higher in females than in males, and speculated that the results may be related to hormonal changes that occur during menopause.^11^ G17 is unstable and its expression level is affected by many factors (regions, lesion location, atrophic degree, etc.). This study verified that both SAA and *H. pylori* infection can impact on the secretion of G17.

*H. pylori* is considered as a primary carcinogen.^12^ According to the studies about *H. pylori* infection carried out in China, in *H. pylori*‐positive patients, the serum levels of G17, PGI and PG II increase, especially PGII, while the ratio of PGI/PGII decreases.^13^ This study identified that the level of G17 in *H. pylori*‐positive subjects was remarkably higher than that in *H. pylori*‐negative subjects, coinciding with another study.^14^ Patients with *H. pylori* infection and low PGI/PGII ratio are confronted with a higher risk of gastric cancer, and thus secondary diagnosis (endoscopy and histology) should be performed.^15^

Further, the combination of serum PG and G17 levels can help diagnose gastric mucosa, reflect the degree of gastric aging, and distinguish pathological state from healthy state.^9^ A study showed that the combination of G17 and different serum markers can improve the diagnostic efficiency of gastric cancer.^8^ PG is the precursor of pepsin, which is mainly secreted by the gastric chief cells. PGI, PGII and their ratio are considered as predictors of various gastric diseases, including AG and IM which are defined as precancerous lesions of gastric cancer.^16^ PG tests showed poor sensitivity (range: 18.4–40.5) but high specificity (>93%) to advanced precancerous lesions. In this study, G17 and PG exhibited a weak to moderate correlation, consistent with Wang Rui's results,^17^ which mirrored that the combination of G17 and PG can exert a better screening effect.

Therefore, G17 is not the major diagnostic indicator, but can be combined with PG and *H. pylori* detection as reference to further improve the diagnosis rate of AG and early gastric cancer, and also to directly screen high‐risk groups. On the basis of G17, PG, and *H. pylori* detection results, endoscopy and histological examination were scheduled to enhance the compliance of screening and treatment of gastric diseases, and avoid unnecessary radiation and discomfort caused by endoscopy. Besides, former findings have unveiled that G17 combined with serum tumor markers (CEA, CA12‐5, and CA19‐9) can significantly improve the sensitivity of the diagnosis of gastric cancer.^18^

In recent years, Chinese scholars have successively promoted the “four items for gastric function” test combined with G17, PG, and *H. pylori*, based on the overseas screening mode of gastric cancer and combined with the situation of China and the current epidemiological investigation. Through this test, it is expected to find and optimize the early gastric cancer screening method suitable for China's national conditions, and timely provide evaluation and intervention recommendations for high‐risk groups. Also, it is hopeful to reduce mortality, prolong life, and protect people's health by early screening, early diagnosis and early treatment. Nevertheless, in fact, some limitations exist in our study. Reportedly, several separate and combinatorial transcripts of *H. pylori* virulence genes are associated with clinical outcomes (gastritis, gastric cancer, tumor grade, and tumor area).^19^ However, our study did not involve this aspect. Therefore, the relationship between *H. pylori* genotype and G17 level needs further study. Besides, the grading and staging of gastric cancer also need to be examined to confirm the relationship between G17 level and gastric cancer.

## 5 CONCLUSION

Since China is challenged by high incidence rate and mortality rate of gastric cancer, and precancerous lesions are the key stage in the development of gastric cancer, it is necessary to find effective means to screen gastrointestinal diseases. Combined screening is conducive to early screening gastrointestinal diseases in normal people or groups at high risk of gastric cancer, but the influence of relevant factors on G17 should be excluded to enhance the reliability of the results.

## AUTHOR CONTRIBUTIONS

**Junchao Zeng**: conceptualization; writing—original draft. **Yan Shen**: project administration; writing—review & editing. **Sanping Xu**: formal analysis; investigation; resources; software. **Rui Yang**: investigation; validation; visualization.

## CONFLICT OF INTEREST STATEMENT

The authors declare no conflicts of interest.

### PREPRINT

A preprint has previously been published.^20^

## Data Availability

The analyzed datasets generated during the study are available from the corresponding author on reasonable request.
